# Prevalence and spectrum of germline BRCA1 and BRCA2 in a cohort of ovarian cancer patients from the Salento peninsula (Southern Italy): a matter of preventive health

**DOI:** 10.18632/oncotarget.28561

**Published:** 2024-02-22

**Authors:** Elisabetta De Matteis, Maria Rosaria Tumolo, Paolo Tarantino, Mariangela Ciccarese, Tiziana Grassi, Francesco Bagordo, Maria Rita De Giorgio, Emanuele Rizzo, Graziana Ronzino

**Affiliations:** ^1^U.O.S.V.D. Oncological Screenings, “Vito Fazzi” Hospital, Lecce, Italy; ^2^Department of Biological and Environmental Sciences and Technology, University of Salento, Lecce, Italy; ^3^U.O.C. Medical Genetics, “Vito Fazzi” Hospital, Lecce, Italy; ^4^Strategic Regional Agency for Health and Social of Puglia, Bari, Italy; ^5^Department of Pharmacy-Pharmaceutical Sciences, University of Bari “Aldo Moro”, Bari, Italy; ^6^Oncology Unit, “Vito Fazzi” Hospital, Lecce, Italy

**Keywords:** ovarian cancer, BRCA1, BRCA2, mutation

## Abstract

Objectives: The aim of this exploratory, descriptive study was to characterize the deleterious BRCA1 and BRCA2 variants evaluated by genetic testing in a group of Ovarian cancer patients living in the Salento peninsula (Southern Italy).

Methods: From June 2014 to July 2023, patients with histologically confirmed high-grade serous carcinoma, fallopian tube, or primary peritoneal cancer who were referred to Lecce Familial Cancer Clinic were considered. BRCA-mutation genetic testing was performed on these patients. Socio-demographic data and cancer epidemiology were assessed, and Next Generation Sequencing and Sanger DNA sequencing were performed.

Results: The median age at the diagnosis of 332 ovarian cancer patients collected was 57 years. The pedigree analyses showed that 28.6% had familial cases and 39.7% had sporadic cases. Of the 319 patients submitted to genetic testing, 29.8% were carriers of BRCA1/2 mutation, 75.8% at BRCA1 and 24.2% at BRCA2 gene. Of the 21 BRCA1 mutations, the variant c.5266dupC was the most frequent alteration (28.4%). With respect to BRCA2, 13 mutations were found and the variant c.9676delT was the most frequently recorded (6.3%).

Conclusions: This study reveals that the prevalence of germline mutations in the BRCA1 and BRCA2 genes was higher than reported by other studies. A broader understanding of the prevalence and role of BRCA mutations in development, response to treatment, and prognosis represents an exciting and developing area of ovarian cancer treatment and prevention.

## INTRODUCTION

Ovarian cancer (OC) is the third most prevalent type of gynecologic cancer affecting women after cervical and uterine cancer [1]. It is also considered the most fatal of all gynecological cancers due to the non-specific signs of the disease and the lack of early detection testing so most patients are diagnosed at an advanced stage, which is associated with poor survival [2]. OC is associated with 2.1% of newly reported deaths from all cancer types each year [3]. In Italy, 5,200 new OC cases were estimated in 2020 while 3,200 deaths for OC occurred among women in 2021 [4].

The most frequent type of OC is epithelial OC (EOC), accounting for almost 90% of all OCs [5], and high-grade serous carcinoma (HGSC) represents the most frequent and aggressive histological subtype [6]. HGSC was previously believed to originate from the epithelium of the ovary, while new data indicate that it comes from a precursor within the secretory epithelial cells of the fallopian tube [7, 8].

Although age, family history and lifestyle affect the pathogenesis of OC [9], mutations in BReast CAncer 1 (BRCA1) and BReast CAncer 2 (BRCA2) genes represent the most significant risk factors associated with it [10]. These mutations are also related to a high risk of developing breast, pancreatic, and prostate cancer [11–14]. The lifetime risk of developing OC in women up to age 80 is estimated at 44% for BRCA1 and 17% for BRCA2 mutation carriers [15]. BRCA1/2, located on chromosomes 17q21 and 13q12, respectively, transcribe proteins with a crucial role in the repair of DNA double-strand breaks (DSBs); notably, BRCA1 surveys the DNA for DSBs, and BRCA2, along with other molecules, attach to the damaged site and repair the DNA. Hence, cells containing mutated BRCA1/2 proteins exhibit deleterious (pathogenic) dysfunctions of the homologous recombination process and cannot repair DSBs, ultimately causing genomic instability, cell death, and a high risk of malignant transformation, and thus functioning as tumor suppressor genes [16–18].

Several studies demonstrated that the prevalence of pathogenic variants of BRCA1/2 could change on the basis of geographic distribution, race and ethnicity [19, 20]. Some populations present a wide spectrum of different variants in both the BRCA1 and BRCA2 genes, whereas in other populations, geographically restricted and/or with homogenous genomic characteristics, specific mutations in BRCA1/2 occur with high frequency, as a consequence of a founder effect. This phenomenon is caused by the mating (or breeding) between individuals that are genetically closely related or blood relatives [19].

In a recent study conducted on 1,346 patients with hereditary breast/ovarian cancer, thirty pathogenic variants were identified in the Sicilian population but only some of these showed a specific territorial prevalence, unlike other Italian and European regions. This difference could be attributed to the historical background of the Sicily and genetic heterogeneity of this population [21]. In another study, Rebbeck et al. identified several mutations with relatively high frequency that, in addition to the known founder mutations, well characterized specific racial/ethnic or geographic groups. In particular the most common mutations in Italy were: BRCA1 c.5266dup, c.181T>G, c.190T>C, c.1687C>T, and c.1380dup; BRCA2 c.8878C>T, c.6468_6469del, c. 7180A>T, c. 5682C>G, c. 8247_8248delGA [20]. Loizzi et al. studied the incidence of the BRCA mutations in a population of Apulian OC with a high-grade serous histotype, finding 39% of probands carrying a BRCA 1/2 mutation. This rate is higher than that typically observed in the general Italian population, probably due to a founder effect. The authors suggest that the most representative mutations (BRCA1: c.5263_6264insC, c.65T>C; BRCA2: c.5796_5797delTA) were unique founder mutations in the Apulia region. Moreover, the study demonstrated that BRCA-mutated patients had a lower median age of onset (53 years), a lower rate of advanced stages diagnosis and a lower mortality than wild-type patients, as well as higher mean values of Progression Free Survival and Overall Survival [22].

In the light of these considerations, the knowledge of the population-specific mutation spectrum in BRCA1/2 could provide efficient strategies for genetic testing. This is important both for healthy carriers, because they may require more intensive and individualized screening as well as prophylactic surgery, and for cancer patients, because these genetic mutations may lead to potential target therapy (such as poly(ADP-ribose) polymerase inhibitors) [18]. Moreover, it should also be emphasized that, though rapid tests are necessary, onco-genetic counselling is a mandatory and crucial strategy to obtain an accurate study of family history and subsequently plan health intervention both for a prevention strategy in healthy carrier and for the strategy therapeutic in those affected. The interventions of primary prevention in the families of these individuals are pivotal, considering the lethality of the disease and the lack of adequate screening protocols. Currently primary prevention is a public health issue and a way to empower the sustainability of the system in Italy which has a National Health Service that provides universal coverage to all citizens and legal foreign residents [23].

The aim of this exploratory study was to characterize the deleterious BRCA1 and BRCA2 variants evaluated by genetic testing in a group of OC patients living in the Salento peninsula (Southern Italy).

## RESULTS

Between June 2014 and July 2023, a total of 332 OC patients, who met the previously established criteria, were studied. The median age at the diagnosis was 57 years (range 25–91 years).

The pedigree analyses showed that 95 (28.6%) out of 332 had familial cases and 132 (39.7%) had sporadic cases. The most common types of familial cancer reported were BC cancer (179 cases) and OC cancer (56 cases).

The histological tumor subtypes were distributed as follows: 245 high-grade serous ovarian adenocarcinoma (HGSP) (73.8%), 30 endometrioid (9%), 16 clear cell (4.8%), 34 other/not available (10.2%), 4 borderline (1%), and 3 mucinous (0.9%). Patient characteristics were listed in Table 1 (Table 1).

**Table 1:** Characteristics of ovarian cancer patients (*n* = 332)

Characteristics	No. patients (%)
Sporadic cases	132 (39.7%)
Familial cases	95 (28.6%)
Ovarian cancer patients with family history of:	
*Ovarian cancer*	56 (16.8%)
*Breast cancer*	179 (53.92%)
*Bilateral Breast cancer*	9 (2.71%)
*Endometrial cancer*	13 (3.92%)
*Prostate cancer*	38 (11.45%)
Histological types	
*High-grade serous ovarian adenocarcinoma*	245 (73.8%)
*Endometrioid*	30 (9%)
*Clear cell*	16 (4.8%)
*Mucinous*	3 (0.9%)
*Borderline*	4 (1.2%)
*Other/not available*	34 (10.2%)
Figo Stages	
*Stage I*	64 (19.2%)
*Stage II*	27 (8.1%)
*Stage III*	123 (37%)
*Stage IV*	47 (14.2%)
*Unknown*	71 (21.4%)

Of the 332 OC patients, BRCA test was performed on 319 patients. The genetic analysis revealed the presence of 6.6% (21/319) variants of uncertain significance (VUS), while germinal BRCA mutations were detected in 29.8% (95/319) of the patients; of these, 75.8% (72/95) were BRCA1, and 24.2% (23/95) were BRCA2 mutations.

A total of 34 mutations were detected in the BRCA genes, specifically 21 in BRCA1 and 13 in BRCA2. The most common mutation for BRCA1 carrier patients was c.5266dupC, reported in 28.4% (27/95) of patients. Among BRCA2 pathogenic variants, c.9676delT was the most prevalent (6.3%). In Table 2 all the PV variants found in the analyzed patients were reported (Table 2).

**Table 2:** BRCA1/2 mutations in the analyzed population

Genes	HGVS^1^ cDNA	HGVS^1^ protein	Exon/intron	Molecular consequences	Classes	No. of carriers
BRCA1	c.287A>G	p.(Asp96Gly)	5	missense	4	1
	c.(?_−232)_(80+1_81–1)del	Ex. 1-2del	1–2	CNV^2^	5	4
	c.1375A>T	p.(Lys459Ter)	11	nonsense	5	1
	c.1504_1508del	p.(Leu502Alafs^*^2)	11	frameshift	5	4
	c.1569delinsATCAAGAAA	p.(Ala524Serfs^*^11)	11	frameshift	5	1
	c.1687 C>T	p.(Gln563^*^)	11	nonsense	5	1
	c.181T>G	p.(Cys61Gly)	5	missense	5	4
	c.1887_1900dup	p.(Pro634delinsGlnIle^*^)	11	frameshift	5	2
	c.190T>C	p.(Cys64Arg)	5	missense	5	1
	c.2079_2080delCA	p.(Asp693Glufs)	11	frameshift	5	1
	c.212+2T>A	p.(?)	5	splicing	5	1
	c.2982_2986delTCATC	p.(Ser956Valfs^*^13)	11	frameshift	5	1
	c.3779_3771delAC	p.(Glu1257Glyfs^*^9)	11	frameshift	5	1
	c.4302C>T	p.(Gln1395^*^)	12	nonsense	5	1
	c.4327 C>T	p.(Arg1443^*^)	13	nonsense	5	4
	c.4576G>T	p.(Glu1526^*^)	15	nonsense	5	1
	c.4999A>T	p.(Lys1667^*^)	17	nonsense	5	1
	c.514del	p.(Gln172fs)	8	frameshift	5	8
	c.5266dupC	p.(Gln1756Profs)	19	frameshift	5	27
	c.5278-2del	p.(?)	2	splicing	5	1
	c.65T>C	p.(Leu22Ser)	2	missense	5	6
BRCA2	c.1796_1800delTTTAT	p.(Ser599^*^fs)	1	frameshift	5	1
	c.1832C>A	p.(Ser611^*^)	10	nonsense	5	1
	c.2808_2811delACAA	p.(Ala938Profs)	11	frameshift	5	1
	c.3036_3039delACAA	p.(Ala938Profs^*^21)	11	frameshift	5	1
	c.4512dupT	p.(Gln1429Serfs^*^9)	11	frameshift	5	1
	c.4525C>T	p.(Gln1509^*^)	11	nonsense	5	1
	c.5062 G>T	p.(Glu1688^*^)	11	nonsense	5	3
	c.517-3del	p.(?)	6	splicing	5	1
	c.7007G>C	p.(Arg2336Pro)	13	missense	5	1
	c.7251_7252del	p.(His2417Glnfs^*^3)	14	frameshift	5	4
	c.8755-1G>A	p.(?)	21	splicing	5	1
	c.9676delT	p.(Tyr3226Ilefs^*^23)	27	frameshift	5	6
	c.9871delT	p.(Ser3291Leufs^*^21)	27	frameshift	5	1
Total						95

Legend: ^1^HGSV nomenclature, Human Genome Variant Society; ^2^CNV: copy number variation.

## DISCUSSION

It is well known that the presence of mutations in the BRCA1 and BRCA2 genes increases the risk of developing OC [10]. Given the lethality of this disease, the identification of BRCA variants has a relevant impact on prevention, and genetic testing is recommended in eligible women in order to identify those at increased risk of developing OC and allow appropriate prophylactic strategies. Furthermore, genetic testing is critically important for therapy, as it identifies OC patients who can be treated with PARP-inhibitors [24].

In women carrying a germline pathogenic variant in BRCA1/2, there are several risk management strategies recognized as effective and cost-effective to mitigate the increased cancer risk, such as Risk-reducing salpingo-oophorectomy for the prevention of OC [25]. The adoption of these cancer risk management strategies by BRCA carriers remains suboptimal [26].

Furthermore, despite international guidelines for cancer risk management strategies being relatively consistent, substantial variation still exists when it comes to clinical plans for long-term care once a carrier has been identified [27]. In this regard, in the study of Petelin et al. [27] has been found that the long-term management through a structured multidisciplinary familial cancer service is clinically effective and cost-effective for BRCA1/2 carriers.

This observational study investigated the prevalence and spectrum of germline pathogenic variants in BRCA1 or BRCA2 genes in OC patients come from a limited geographical area with presumed homogenous ethnic and genetic characteristics.

The results showed the presence of BRCA1 and BRCA2 mutations in a group of 332 OC patients living in the Salento peninsula (Southern Italy). HGSP (245 cases) was the most represented histology, followed by endometrioid carcinoma (30 cases) and clear cell (16 cases).

The genetic analysis identified 29.8% of BRCA mutations, indicating that the Salento peninsula had a significant mutation incidence, higher than that reported in other studies [28, 29]. Moreover, in line with the literature [22, 28], we found that the majority of patients were BRCA1 carriers (75.8%), confirming that BRCA1 is the most present variant.

The finding of a small percentage of VUS was concordant with data previously reported in the literature [30]. As well known, VUS is a nucleotide sequence alteration with unknown consequences on the possible loss of function of the gene product or on the potential risk of causing disease. Consequently, unclear clinical significance leads to a difficult clinical management by the oncologist and cannot be easily explained to the patient, since a VUS also exhibits a probability of being reclassified as pathogenic between 5 and 94.9% [31].

A total of 34 mutations were detected in the BRCA genes, specifically 21 were BRCA1 mutated and 13 were BRCA2 mutated. The most common mutation for BRCA1 carrier patients was c.5266dupC, reported in 28.4% of our patients. c.5266dupC is one of the three most recorded founder mutations in the Ashkenazi Jews, originating probably more than 38 generation ago from a single founder and spreading both to North America and Europe. For this mutation the lifetime risk of OC is 33%, being the highest among the Jewish founder mutations. This mutation represents the most frequent in Italian population, and the percentage we found is also higher than in other Italian studies [29, 32]. Another variant (c.1375A>T) was described as a founder mutation for Castrignano del Capo (a small city in Apulia) in the study of Capoluongo et al. [33]. c.1375A>T was reported as pathogenic (Class 5; reviewed by Enigma Consortium in the year 2016) in the ClinVar database.

Among BRCA2 pathogenic variants, c.9676delT was the most prevalent one (6.3%). This pathogenic mutation, located in coding exon 26 of the BRCA2 gene, was reported both in the study by Patruno et al. [34] and in that of Santonocito et al. [35]. In the former, the authors analyzed the prevalence of pathogenic germline BRCA1/2 variants in families from Apulia and evaluated the genotype–phenotype correlations [34]. In the latter, the variant was found in patients with breast and/or ovarian cancer admitted to the Oncology Hospital of Rome, a reference center for many patients from Puglia, especially from Lecce [35]. Therefore, it could be assumed that this mutation is of Apulian origin. Future studies will have to confirm this hypothesis.

In conclusion, despite this study having a limited sample size and descriptive design, it allowed to evaluate and describe the frequency of BRCA1 and BRCA2 mutations in a population-based series of cases with ovarian cancer from Salento that is a large area that includes more than 1 million inhabitants. This was a preliminary study which will be followed by further investigations regarding the clinical characteristics of tumors and their correlation with specific variants, as well as the contribution of other risk factors, including additional genetic and environmental factors. A broader understanding of the prevalence and role of BRCA mutations in development, response to treatment, and prognosis represents an exciting and developing area of ovarian cancer treatment and prevention.

It represents a public health issue in a system in which the sustainability of the cost of drugs is in crisis and in which the system’s mission is to reduce the number of people affected and not to treat patients suffering from BRCA disease.

## MATERIALS AND METHODS

### Study design and population

An observational study was performed to describe the prevalence and spectrum of germline BRCA1 and BRCA2 genetic variants in OC patients come from a limited geographical area with presumed homogenous ethnic and genetic characteristics.

Patients with histologically confirmed HGSC, fallopian tube, or primary peritoneal cancer who were referred to Lecce Familial Cancer Clinic between June 2014 to July 2023, were considered for the analysis. These patients were asked to: (1) provide written informed consent; (2) provide any medical records; (3) complete a study questionnaire, which included socio-demographic data and cancer epidemiology; (4) attend a genetic counseling session to discuss family history, allowing the geneticist to trace a three-generation pedigree; and (5) provide a blood sample for DNA extraction and BRCA testing; (6) attend the post-test counselling.

The eligibility to BRCA testing was performed according to criteria reported in the study of Russo and colleagues [36].

### Next-generation sequencing and sanger sequencing analysis for BRCA 1/2 genes

Peripheral blood samples were collected from all patients tested for germline (and if necessary somatic) BRCA1/2 mutations. Genomic DNA was isolated using the High Pure PCR Template Preparation Kit (ROCHE, Basel, Switzerland) while somatic DNA by QIAamp DNA FFPE Tissue Extraction kit (QIAGEN, Hilden, Germany). Samples were quantified by NanoDrop 1000 Spectrophotometer or Qubit4.0 fluorometer (Thermofisher Scientific, Waltham, MA, USA). A mutational analysis of exons and adjacent intronic regions of BRCA1/BRCA2 genes was performed with sanger and next-generation sequencing (NGS).

Sanger testing was conducted using BigDye Therminator 1.1 Cycle Sequencing Kit (Life Technologies, Carlsbad, CA, USA) and read through the 3100 × l Genetic Analyzer (Applied Biosystems, Waltham, MA, USA), according to the manufacturer’s protocols. Devyser BRCA NGS (DEVYSER, Stockholm, Sweden) kit was used to detect both germline and somatic mutations by MiSeq NGS platform (ILLUMINA, San Diego, CA, USA).

### Variant classification

The variant classification was performed according to the criteria adopted by Italian Association of Medical Oncology (AIOM) and proposed by the Evidence-based Network for the Interpretation of Germline Mutant Alleles (ENIGMA) consortium (https://enigmaconsortium.org) which, following the International Agency for Research on Cancer (IARC) recommendations, identifies five classes: Benign (class I), Likely Benign (class II), Variant of Uncertain Significance (VUS, class III), Likely Pathogenic (class IV), and Pathogenic (class V) [37, 38].

ClinVar was used as reference database to categorize the variants as pathogenic [39].

### Statistical analyses

Descriptive analysis using tables, frequency (%), and median values were used to summarize and analyze the data.
